# Air Pollution Monitoring and Mining Based on Sensor Grid in London

**DOI:** 10.3390/s8063601

**Published:** 2008-06-01

**Authors:** Yajie Ma, Mark Richards, Moustafa Ghanem, Yike Guo, John Hassard

**Affiliations:** 1 Department of Computing, Imperial College London, 180 Queens Gate, London SW7 2BW, United Kingdom; E-mail: yajie.ma@imperial.ac.uk; m.ghanem@imperial.ac.uk; y.guo@imperial.ac.uk; 2 Department of Physics, Imperial College London, 180 Queens Gate, London SW7 2BW, United Kingdom; E-mail: mark.richards@imperial.ac.uk; j.hassard@imperial.ac.uk

**Keywords:** urban air pollution, sensor network, grid, distributed data mining

## Abstract

In this paper, we present a distributed infrastructure based on wireless sensors network and Grid computing technology for air pollution monitoring and mining, which aims to develop low-cost and ubiquitous sensor networks to collect real-time, large scale and comprehensive environmental data from road traffic emissions for air pollution monitoring in urban environment. The main informatics challenges in respect to constructing the high-throughput sensor Grid are discussed in this paper. We present a two-layer network framework, a P2P e-Science Grid architecture, and the distributed data mining algorithm as the solutions to address the challenges. We simulated the system in TinyOS to examine the operation of each sensor as well as the networking performance. We also present the distributed data mining result to examine the effectiveness of the algorithm.

## 1. Introduction

Transport has a significant impact upon the environment in which we live. In general, these impacts can be divided under four broad headings: local air quality, climate change, noise and watercourse pollution [1], while the clean air is vital to human health. High levels of fine particulate (PM_10_) air pollution in 2005 were estimated to have caused 1,031 accelerated deaths and 1,088 respiratory hospital admissions in London [2]. The Mayor's Air Quality Strategy [3] was published in 2002 to deal with local air quality and its impact on health. And The Control of Dust and Emissions from Construction and Demolition Guidance [4] was issued in 2006. These documents are used to manage the complex issues of air pollution in London and to develop the London Olympic facilities for 2012 with the minimal impact on London's environment.

However, the volumes of particles and the oxidation of nitrogen in London are still higher than the limitations declared in the Air Quality Standards [5]. The major source of air pollution in London is road traffic emissions. The Environment Agency estimates that traffic sources account for over 97% of CO and 75% NO_X_ emissions. Other notable contributions come from industrial plant and premises, domestic energy production, and construction activity.

In order to monitor the pollutants and analyze their effects to the environment, we developed Mobile Discovery Net (MoDisNet in short) to collect real time pollution data on key aspects of traffic conditions, emissions, ambient pollutant concentration and human exposure. The purpose is to develop the capability to measure, model and predict a wide range of environmental pollutants and hazards using a grid of pervasive roadside and vehicle/person-mounted sensors.

Developing a sensor network over a target region will face a lot of challenges. These include developing and extending existing e-Science Grid, sensor units, communication and modeling technologies to enable the integrating of data from heterogeneous fixed and mobile environmental sensors grids in real time to provide dynamic estimates of pollutants and hazard concentrations; demonstrating how these can be usefully correlated with a wide range of other complementary dynamic data, such as weather, health or traffic data.

In the remainder of this paper, we first describe the motivations for the development of MoDisNet system as well as the main contributions of this paper. Then we discuss the novel techniques we provide to address the problems when a sensor grid is constructed based on the mobile and high-throughput real-time data environment. We also present the system architecture to meet the demands of the project as well as the sensor unit itself. This is then followed by the simulation platform design and the networking performance simulation as well as the real-time pollution data analysis scenarios. We conclude the paper with a summary of the research and a discussion of future work.

## 2. Motivations and Contributions

**Figure 1.**
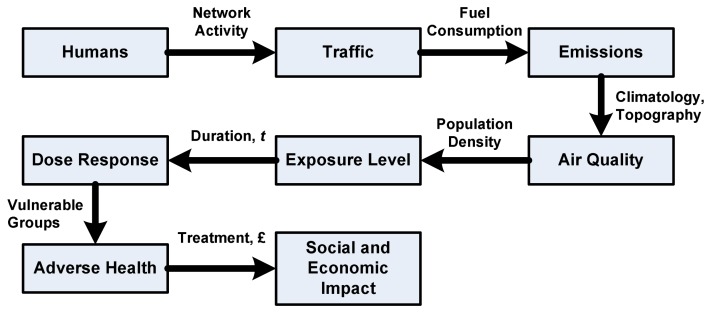
The adverse health impacts chain.

Road traffic makes a significant contribution to the following emissions of pollutants: benzene(C_6_H_6_), 1,3∼butadiene, carbon monoxide(CO), lead, nitrogen dioxide(NO_2_), Ozone(O_3_), particulate matter(PM_10_ and PM_2.5_) and sulphur dioxide(SO_2_). The impact of local air quality pollutants on the environment and health have been studied and well documented [6]. We summarize the interaction and cooperation chain of the population, traffic, air quality and health as Figure 1.

The figure shows that, increased car ownership and use in urban areas (road traffic) generate some chemical emissions to the air to form the air pollution. With various weather conditions (effected by the temperature, wind, humidity, pressure, etc.), these pollutants pose different air qualities. When human beings expose to the polluted air (especially in the urban areas), driving in heavy traffic, near the highways or at the ‘downwind’ locations, with the dose-response, people may suffer breathing problems and asthma attacks, which will contribute to risk of heart attacks among people with heart disease.

Under the current Environment Act of UK [7], most local authorities have air quality monitoring stations to provide environmental information to public daily via internet. To date, the development of work in these areas has been hampered by critical data gaps and asymmetries in data coverage, as well as the lack of on-line data processing capability offered by the e-Science. Information on a number of key factors such as individual driver/vehicle activity, pollution concentration and individual human exposure has traditionally either simply not been available or only available at high levels of spatial and temporal aggregation, which average out scientifically critical local variations. For example, the conventional approach to assessing pollution concentration levels is based on data collected from a network of permanent air quality monitoring stations.

**Figure 2.**
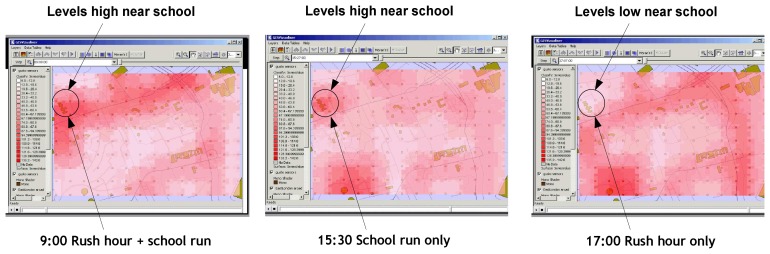
Pollution levels change at East London during a day.

However, permanent monitoring stations are frequently situated so as to measure ambient background concentrations or at potential ‘hotspot’ locations and are usually several kilometers apart. According to our earlier research of ‘Discovery Net EPSRC e-Science Pilot Project’ [8] (the data generated from statically located urban pollution monitoring sensors), we learnt that the pollution levels and the hot spots change with time as shown in Figure 2. However, those results are all computed offline and can't give a real-time track. As the result, it can't make a prompt feedback or supervision to individuals and the air pollution monitor systems.

Besides, while traffic monitoring systems provide information on aggregate traffic parameters, they do not inform on individual vehicle trajectories, in particular, key features (for emissions estimation) such as the incidence of acceleration, deceleration and idling episodes. Moreover, epidemiological studies typically base estimates of exposure on home post code, despite the fact that many people clearly spend large parts of the day in other locations. These data gaps have led to a number of critical barriers to the successful development of key research issues. These include:

1. Model validation: Conventional environmental data sources do not provide a sufficient detail of temporal or spatial resolution to enable existing or emerging traffic, vehicle emissions and pollution dispersion models to be validated at the micro-scale, especially at street level. This is inhibiting the development of necessary understandings of how best to design local traffic management and urban design interventions to reduce pollution concentrations in critical areas, including pollution ‘hot spots’.
2. Human exposure: Little or no data are available at the disaggregate level on individual exposure to pollutants, which is similarly inhibiting the development and validation of exposure modeling. This has important implications, not only scientifically but also practically, especially in terms of demonstrating compliance with existing and future regulatory obligations.
3. Integrated traffic and environmental control: Existing traffic monitoring systems enable adaptive traffic control systems such as SCOOT [9] to draw on real time information on aggregate traffic parameters in order to dynamically optimize network performance to reduce delays (by adjusting signal timings and related measures). The extension of these techniques to the joint optimization of both traffic and environmental outcomes is highly desirable, but currently impossible because of the lack of comparable real-time pollution concentration data.

We can address these concerns by two ways: generating new forms of data (e.g., on exposure and driver/vehicle activity) and generating data at higher levels of spatial and temporal resolution than existing sensor systems. Taking advantage of the low cost mobile environmental sensor system, the MoDisNet system will construct a Grid environment which fully integrates existing static sensor systems and complementary data sources with the mobile environmental sensor system, which will provide radically improved capability for the detection and monitoring of environmental pollutants and hazardous materials.

The main contributions of this paper are: first, we propose a highly effective air pollution monitoring system which fully considers the urban background and the pollution features. In this system, a hierarchical network architecture formed by the mobile sensors and stationary sensors is designed, which makes full use of the roadside devices to fix the stationary sensors as well as the public vehicles to carry the mobile sensors; a ultra violet sensor unit GUSTO which can realize up to 1Hz data collection frequency with high accuracy and low unit cost; a sensor grid framework to provide the processing, integrating, and analyzing heterogeneous sensor data in both centralized and distributed ways. Second, we provide a solution of executing the real-time distributed data mining in sensor grid; design a distributed P2P clustering algorithm for MoDisNet system. Our result also provides a typical air pollution pattern in urban environment which gives a real-time track of the air pollution variation. The result also presents important information about environmental protection and individual supervision.

## 3. Air Pollution Monitoring System Infrastructure

The key feature of the MoDisNet system is to use a variety of vehicle fleets including buses, service vehicles, taxis and commercial vehicles a platform for environmental sensors. With the collaboration of the static sensors fixed on roadside, the whole system can detect the real-time air pollution distribution in London. We will describe the MoDisNet architecture in the following sections in details.

### 3.1. MoDisNet Network Architecture

**Figure 3.**
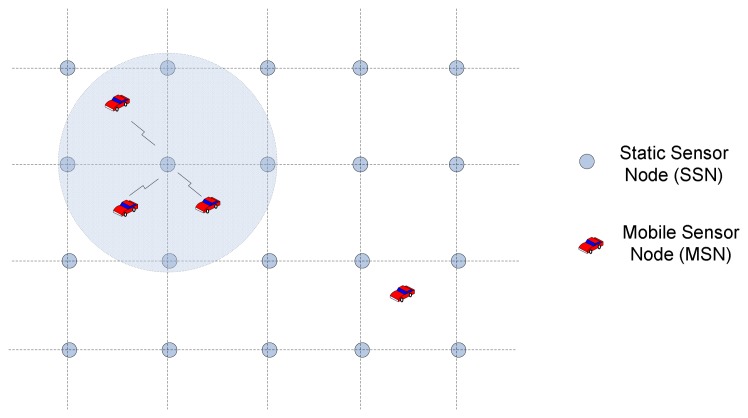
The network architecture of MoDisNet.

The MoDisNet system is constructed based on a novel network environment, which is designed as a two-layer network architecture – the mobile sub-network formed by the Mobile Sensor Nodes (MSN in short) and the stationary sub-network organized by the Static Sensor Nodes (SSN in short). The network architecture is shown in Figure 3.

Here, MSNs are installed in the vehicles. They sample the pollution data and execute the AD conversion to get the digital signals. According to the system requirements, the MSNs may pre-process the raw data (such as the noise reduction, local data cleaning and fusion, etc.) and then send these data to a nearest SSN. The SSNs take in charge of the data receiving, update, storage and exchange works. Cooperating with the e-Science Grid architecture (which will be discussed in detail in section 3.3), the SSNs can realize the distributed data analysis and mining. According to different requirements from the users or the server, the SSNs may send the raw air pollution data or the distributed mining results to the central data warehouse for further process.

### 3.2. GUSTO Sensor Unit

The sensors (including MSN and SSN) we designed within MoDisNet are GUSTO sensor units. GUSTO is an acronym for Generic Ultra violet Sensor Technologies and Observations. It is designed to quantify relative concentrations of a suite of urban air pollutants in real time. The key features of the GUSTO unit are:

1. Simultaneous detection of multiple species of pollutants (SO_2_, NO_X_, O_3_, Benzene and others)
2. Real time data collection and transmission (sampling frequency is approximately 1Hz)
3. Relative low unit cost (compared to permanent monitoring sites)
4. Robust (self corrects for background changes for each scan)
5. Accurate over ambient concentrations (ppb levels)

GUSTO makes use of the characteristic narrow band absorption of the gas under study (includes SO_2_, NO, NO_2_, O_3_, NH_3_, and Benzene) in the UV spectral range 200-300nm. Retrievals are based on a variation of the well established Beer-Lambert Law, which describes the empirical relationship that relates the absorption of light to the properties of the material through which the light is traveling. Accordingly, the amount of light emerging from a sample is diminished by three physical phenomena:

1. The amount of absorbing material in its optical path (concentration)
2. The distance the light must travel through the sample (path length)
3. The probability that the photon of that particular wavelength will be absorbed by the material (absorptivity or extinction coefficient)

This relationship can be expressed mathematically and has been covered in several previous works [8].

**Figure 4.**
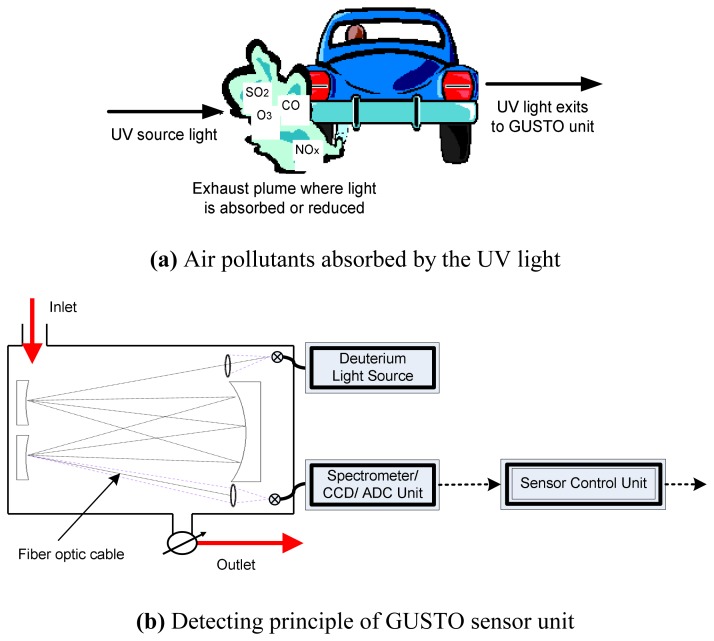
GUSTO sensor unit schematic representation

A schematic of the GUSTO unit for deployment within the MoDisNet program is presented in Figure 4. When vehicles pass within the vicinity of the sensor, key pollutants (SO_2_, NO_X_, O_3_, etc) emitted by such vehicles absorb UV light at characteristic frequencies and then detected by the GUSTO sensors as illustrated in Figure 4(a).

Figure 4(b) shows that the sensor unit primarily consists of four main components: (1) the Deuterium Light Source (DLS), (2) UV optics in the form of a Multi-pass White Cell (MWC), (3) a Spectrometer and a Linear CCD unit, and (4) the sensor control unit for data processing and transmission. The sensor is closed path and ambient air is sampled at a frequency of around 1Hz. The UV light from the DLS passes through the Deuterium and the resulting spectral output is directed along an open optical path via a set of Vertical Transfer Mirror. Then the spectral output imaged onto the surface of the CCD detector. The intensity values are obtained via a 14-bit ADC to produce an atmospheric spectrum of wavelength versus intensity over the GUSTO range. The narrow absorption features are subsequently de-convolved from the atmospheric spectrum and the resulting differentials are used to calculate the concentration of each absorber.

### 3.3. e-Science Infrastructure Based on Grid Computing

#### 3.3.1 e-Science and Grid

Today's research depends increasingly on communication and cooperation. More and more, scientists need to share resources through distributed computing and databases, gaining access to specialized and expensive facilities by developing national and international collaborations. Often, this involves integrating complex data repositories, terascale computing and high performance visualization now available in many research areas.

The term Enhanced Science, or e-Science is ‘refer to the large scale science that will increasingly be carried out through distributed global collaborations enabled by the Internet. Typically, a feature of such collaborative scientific enterprises is that they will require access to very large data collections, very large scale computing resources and high performance visualization back to the individual user scientists.’ [10]

In this description, as in many others, e-Science is closely associated with Grid computer network architecture that enables much of the global collaboration considered basic to e-Science [11, 12]. The Grid is an architecture proposed to bring all these issues together and make a reality of such a vision for e-Science. Ian Foster and Carl Kesselman, inventors of the Globus approach to the Grid, define the Grid as an enabler for Virtual Organizations: ‘An infrastructure that enables flexible, secure, coordinated resource sharing among dynamic collections of individuals, institutions and resources.’ It is important to recognize that resource in this context includes computational systems and data storage and specialized experimental facilities [10].

Currently, some research groups are working on the e-Science architecture design and development, including the TIME-EACM [13] project based at the University of Cambridge Computer Laboratory, and the North East Regional e-Science Centre (NEReSC) [14] based at the University of Newcastle, etc. Most of their researches address the issues of real-time data query, distributed data access and heterogeneity management.

However, MoDisNet aims to develop and deliver a making system for pervasive mobile environmental sensors. The work is based on developing a ‘Mobile Sensor Data Grid’ for processing, integrating, and analyzing heterogeneous sensor data. Based on the former research ‘Discovery Net EPSRC e-Science Pilot Project’ [8], we have developed a service-based infrastructure for scientific informatics that supports the analysis of data generated from statically located urban pollution monitoring sensors. However, the support of a high-number of mobile sensors within a dynamic environment presents new challenges to Discovery Net. The number and nature of such sensor networks mean that in most instances the data cannot realistically be warehoused and then analyzed off-line. A new paradigm is required where much of the analysis is performed within the network itself using pervasive computing technologies such as the Peer-to-Peer (P2P) model [15]. This is feasible as even rudimentary sensors will have processing capability and sensor-to-sensor protocols can be extended to support the dynamic real-time execution of analysis and mining algorithms.

#### 3.3.2 P2P-based Sensor Grid architecture in MoDisNet

Within large scale mobile sensor network architectures, the sensors themselves naturally form and communicate with each other as a P2P network. As the GUSTO sensor can measure pollutants at very high level of accuracy and throughput at very short intervals, which means the volumes of generated and transferred data can be up to gigabit magnitude each day per sensor. This raises many informatics challenges to the data process and storage. In order to satisfy the real-time analysis requirements, the sensors themselves must store part of the information and communicate with each other within a P2P network. The measurements from sensors, both mobile and static, will be filtered and processed using a set of specialized algorithmic processes, before being warehoused within a repository. In order to satisfy the real-time analysis requirements as well as the data storage/communication trade-offs, the sensors in MoDisNet Grid are equipped with sufficient computational capabilities to participate in the Grid environment and to feed data to the warehouse as well as perform analysis tasks and communicating with their peers.

**Figure 5.**
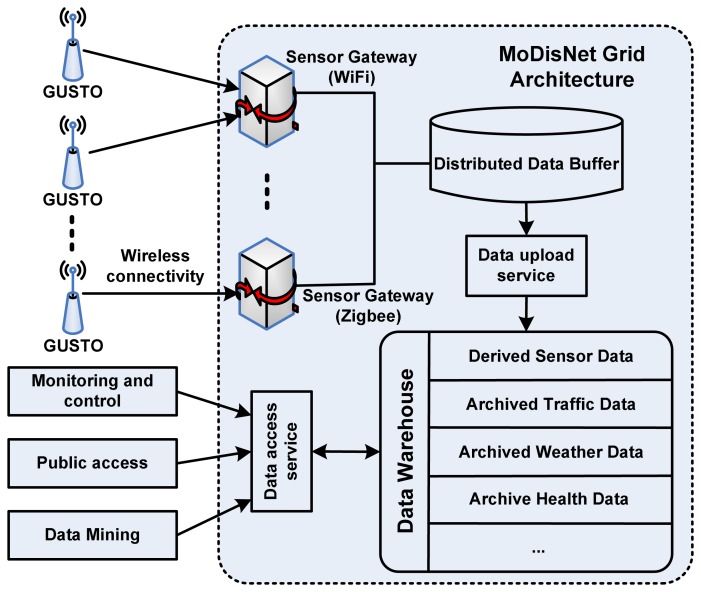
The MoDisNet sensor grid architecture.

Within MoDisNet the sensor grid should be developed to support two important techniques: firstly the techniques allowing the analysis of transport, weather and pollution data in real-time using P2P methods and protocols; secondly the design of new communication protocols supporting dynamic real time data aggregation and statistics. These will provide MoDisNet with the ability to support the full scale analytical task ranging from dynamic real time mining of sensor data to the analysis of off-line data warehoused for historical analysis. To satisfy the demands above, the MoDisNet sensor grid architecture is designed as illustrated in Figure 5.

The GUSTO sensors (including SSN and MSN) connect to the MoDisNet Grid by several Sensor Gateways (SGs) according to different wireless access protocols. The sensors are capable of collecting the air pollution data up to 1Hz frequency and sending the data to the remote Grid service hop by hop (a multi-hop style). This capability enable the sensors exchange their raw data locally and then realize the data analysis and mining in distributed way. This capability also presents the potential for further data fusion and aggregation (which is beyond the research of this paper, and we will discuss it briefly in section 7). The SGs take in charge of connecting the wireless sensor network with the IP backbone, which can be either wired or wireless. These SGs can monitor the volumes of the data streams from the sensors and execute the load balancing function to avoid transfer collisions, which is very useful for improving the throughput and performance of the Grid architecture. A warehouse that can be accessed by SQL database is managed by the Grid architecture which centrally stores and maintains all the archived data, including derived sensor data and the third part data such as the traffic data, the weather data and the health data. These data can provide wealth of information for the Grid computation to generate the short-term or long-term models that relate to the air pollution and traffic, down to the level of streets and buildings. Further more, it may give the supervision for the prediction of the forthcoming events about the traffic change and pollution trend. In term of the visual workflow tools, it can also provide real-time output to the end user to make a full understanding of the air pollution and traffic conditions in different locations within the monitor areas.

## 4. Data Mining

### 4.1 Data Mining Requirements within MoDisNet

Within the MoDisNet Project, a substantial level of resource has been assigned to the development of a set of data mining techniques specific to the needs of research into the relationships between urban transport and the environment. In this system, we can define the data mining tasks by three aspects:

#### 4.1.1 Centralized Data Mining

The data stored within the central data warehouse will often need to be queried by the end-users in order to find regularities in the pollution fluctuations, traffic data and other sensor information. These queries will be easily configurable using Grid service elements within the MoDisNet environment, and potentially published as Grid services themselves. In this scenario, the data mining is necessarily implemented in the warehouse. For the centralized data mining, we seek to identify the long-term patterns of pollution and traffic, and thereby, identify expected baseline conditions and key relationship between certain pollutants and traffic events, etc. This will consist of relatively complex mining processes for expected relationships and techniques to identify potentially new relationships in the data. Due to this complexity and the need to access multiple data archives, each running of the centralized mining may take a long time. It will depend on the amount of the data under consideration and the complexity of the algorithms. As our former project Discovery Net forms an ideal engineering framework for rapidly building up the system, we will not discuss this case in details.

#### 4.1.2 P2P Based Distributed Data Mining

Historical data is well-suited to large-scale analysis over multiple dimensions, but for dynamic queries over real-time sensor data streams, the data has to be taken directly from the sensors. These data points have little value for warehousing and also the real time mining querying cannot afford a “store and mining” model. A typical analytical work would involve the statistics at a certain location and about certain properties in that location. The sensor may not be able to offer this information on its own, due to its movement from the location, or due to inability to capture all relevant information pertaining to the query. The dynamically composed sensor network with P2P communication model can support such information exchange and distributed streaming mining algorithms to provide real time analytical querying model.

The P2P in-networks data mining is based on the data collected and stored by each sensor in real time. Mining these real-time data, the system can give a quick judgment on the pollution status. And the result may reflect some change of the traffic situations. The convergence time of the distributed mining algorithms must be as quick as possible. At the same time, the results may be very simple and not accurate as the centralized one.

#### 4.1.3 Integrated Data Mining

Based on the distributed data mining, we can be aware of some abnormal air pollution conditions. It may trigger the centralized data mining execution in two ways:

- The distributed mining results may match a exist pattern achieved by the centralized mining. So we can suppose that some traffic events may have happened or will happen.
- It is a whole new result that the centralized mining never learnt before. So it may run the centralized algorithm again to re-calculate a new model that is more complete and accurate than the formal one.

In the following sections, we will focus on the distributed data mining (DDM) technique. As described in 4.1.2, this is the key point of the data mining tasks in MoDisNet system. We will first overview the background and recent researches on DDM, and then propose a P2P based distributed clustering algorithm which is designed for pattern recognition of the urban air pollution.

### 4.2 Distributed Data Mining in Sensor Networks

Data mining in sensor networks faces several challenges. First, sensors are seriously constrained by the resource, including battery lifetime, communication bandwidth, CPU capability and storage [16]. Second, sensor node mobility increases the complexity of sensor data collection and analysis [17, 18]. Third, sensor data come in time-ordered streams over network, which makes traditional centralized mining techniques inapplicable. As in MoDisNet system, not only the types of pollutants measured by sensors, but also since sensors may be mobile it is essential to record the locations of the sensors at each measurement time. As the result, mining these data requires a multitude of analysis components such as statistical, clustering, visualization and classification algorithms and tools. Besides, the analysis of spatiotemporal variation of multiple pollutants with respect to one another can be directly performed over the collected pollution data, however the correlation with third-party data, such as weather, health or traffic is more important and needs novel dynamic data access and integration techniques.

For sensor networks, the sensors collect data in time sequence and then there is continuous stream of incoming data for each sensor. Because of the limited storage capability, storing the historical data in each sensor is difficult, even for storing the summary/pattern from the historical data. At the same time, sensor network data management may need the on-line analysis results to be presented for the real-time monitoring and supervising. As the result, the real-time DDM schemes are significantly demanded in such scenario.

DDM offers an alternate approach to address above problems of mining data using distributed resources. DDM pays careful attention to distributed data, computing, communication, and human resources to use them in a near-optimal fashion [19]. In recent years, there are a lot of researches on DDM in sensor networks. The existing work can be arranged into three aspects:

1. intelligent data collection schemes to reduce data volume;
2. optimal nodes organization strategies to eliminate the heterogeneous of sensor data; and
3. highly efficient mining algorithms to reduce the computing complexity of DDM.

For the first scenario, an unsupervised approach to the outlier detection problem in sensor networks is presented in [20], where kernel density estimators are used to estimate the distribution of the data generated by the sensors. [21] studied the problem of detecting regions of interesting environmental events, which assumes that faults can occur in the equipments though they would be uncorrelated, while environmental conditions are spatially correlated. In [22], the authors partition the sensor grid into several subnets. If the density of nodes is high enough, one node in each subnet can be chosen to give an estimate of the samples at every other node within its subnet. As the result, the collected data volumes are highly reduced.

Among the strategies of the second aspect, arranging the nodes of the sensor networks in logical hierarchical styles is a widely used method. In [23], a multi-dimensional clustering approach was proposed to set up a two-layer framework, in which the sensor nodes grouped into cliques on the bottom layer and the data abstraction represents the top layer. Under this framework, a distributed pattern recognition scheme is executed. [24] also proposed a two-layer modular architecture to adaptively perform data mining tasks in large sensor networks. The architecture consists in a lower layer which performs data aggregation in a modular fashion and an upper layer which employs an adaptive local learning technique to extract a prediction model from the aggregated information.

The high efficient mining algorithms can present the distributed mining result directly and many efforts have been made in this area. In [25], an in-network data mining technique to discover frequent event patterns and their spatial-temporal properties was proposed, where each node collects information from its neighbors. [26] presented a self-stabilizing peer-to-peer indexing structure and an efficient nearest neighbor querying methods. [27] worked on the distributed error minimization algorithm for density estimation and clustering in sensor networks. A distributed approach for event prediction was proposed in [28], where each sensor learns to make local decision about capturing the environmental change.

In next section, we will present our distributed data mining algorithm—a P2P clustering algorithm for air pollution pattern recognition which aims to reduce the computing complexity and generate real-time mining results.

### 4.3. Distributed Clustering Algorithm within MoDisNet

To realize the P2P based DDM, the algorithm has to provide the information exchange in P2P style. Here, we designed a clustering algorithm which can satisfy the P2P mining demands. The clustering problem is defined as follow: clustering is the process of grouping the data into classes of clusters so that objects within a cluster have high similarity in comparison to one another, but are very dissimilar to objects in other clusters [29]. Cluster analysis has been widely used in numerous applications, including pattern recognition, data analysis, image regions and market research. We use the clustering analysis in MoDisNet system to find out the pollution patterns (or pollution clouds) in the urban environments. The distributed data process including the data process in both MSNs and SSNs. For the MSNs, the main tasks are:

1. sensing the air pollution data and conveying the analogy signals to digital signals;
2. storing the sampled data; and
3. sending the data to nearest SSN if the timer in MSN expires.

And for the SSNs, the main tasks are:

1. receiving data from MSNs;
2. choosing a certain number of SSNs as the Information Exchange Node Set (IENS) in term of a random algorithm (the random algorithm is beyond the discussion of this paper), then sending polling message to those nodes and waiting for the data exchange messages;
3. receiving the data exchange messages from the SSNs in IENS; and
4. executing the distributed data mining algorithm.

To realize the data processing and analyzing functions described above, a distributed *k*-means algorithm (the goal is to find *k* centers that minimize the maximum distance of a point to its closest center [30]) is designed to mining the air pollution patterns in different locations according to the sampled air pollutants' volumes. This algorithm runs in each SSN. To describe this algorithm, we explain some of the definitions first (suppose the total numbers of SSN is n (n > 0)).

- *SSN_i_*: a SSN node with the identity *i* (*i* = 0, …, *n*−1);
- *S_i_*: an Information Exchange Node Set (IENS) of *SSN_i_*, which is a set of some of the SSNs that can exchange information with *SSN_i_*;
- *k*: the number of clusters that required in *k*-means algorithm (*k* > 0);
- *C^l^_i_*,*_j_*: the cluster center of *j*th (*j* = 0, …, *k*−1) cluster that is computed in *SSN_i_* in *l*th recursion (*l* ≥ 0);
- *Num_i_*,*_j_*: the number of members (data points) belongs to *j*th cluster in *SSN_i_*;
- *δ*: a pre-defined threshold.

The algorithm is described as below:

Node *SSN_i_* chooses a certain number of SSNs as *S_i_* in term of a random algorithm;Node *SSN_i_* receives data from MSNs as the local data and chooses *k* local data as the initial local cluster centres *C*^0^*_i_*,*_j_* (*j* = 0, …, *k*−1);Node *SSN_i_* calculates the Euclidean distance between each local data and each *C*^0^*_i_*,*_j_*;Node *SSN_i_* distributes each data to the nearest *C*^0^*_i_*,*_j_* as the member of this cluster, and each local cluster of *SSN_i_* can be described as (*C*^0^*_i_*,*_j_*, *Num_i_*,*_j_*);Node *SSN_i_* sends polling messages in intervals to each SSN in *S_i_* and expect to receive data information form them;Node *SSN_x_* in *S_i_* sends back the local data description (*C*^0^*_x_*,*_j_*, *Num_x_*,*_j_*) to *SSN_i_* if it receives the polling message from *SSN_i_*;If node *SSN_i_* receives all the data information it expects send back from all the node in *S_i_*, *SSN_i_* calculates the new cluster centres as *C*^1^*_i_*,*_j_*;Node *SSN_i_* computes the offset between *C*^1^*_i_*,*_j_* and *C*^0^*_i_*,*_j_*, if the offset ≤ *δ*, then the algorithm finishes; otherwise replaces *C*^0^*_i_*,*_j_* by *C*^1^*_i_*,*_j_*, and go to step 3.


## 5. System Operational Simulation

### 5.1. Simulation Platform

The operational simulation of the MoDisNet system aims to give an overall evaluation of the hardware and software design. The simulation will realize not only the wireless communication processing and upper layer algorithms, but also the sensor unit itself, including the reasonability of all the function modules of the sensor chip, the lower layer driving programs, and the cooperation of different types of sensors (MSN and SSN). For this purpose, we designed a simulation platform which has the capabilities to simulate all the functions of a single sensor, and the functions of information exchange between different sensors.

To simulate the functions of a single sensor, we designed and coded MSN/SSN units based on the TinyOS system. TinyOS is an open-source operating system designed for wireless embedded sensor networks. It features a component-based architecture which enables rapid innovation and implementation while minimizing code size as required by the severe memory constraints inherent in sensor networks [31]. To realize all the functions that a MSN or SSN requires, we have to choose suitable system components that the TinyOS system provides, as well as design reasonable user/application components based on the functional descriptions listed in section 4.3. Besides, we need to use different interfaces to link all the components to make them work together.

**Figure 6.**
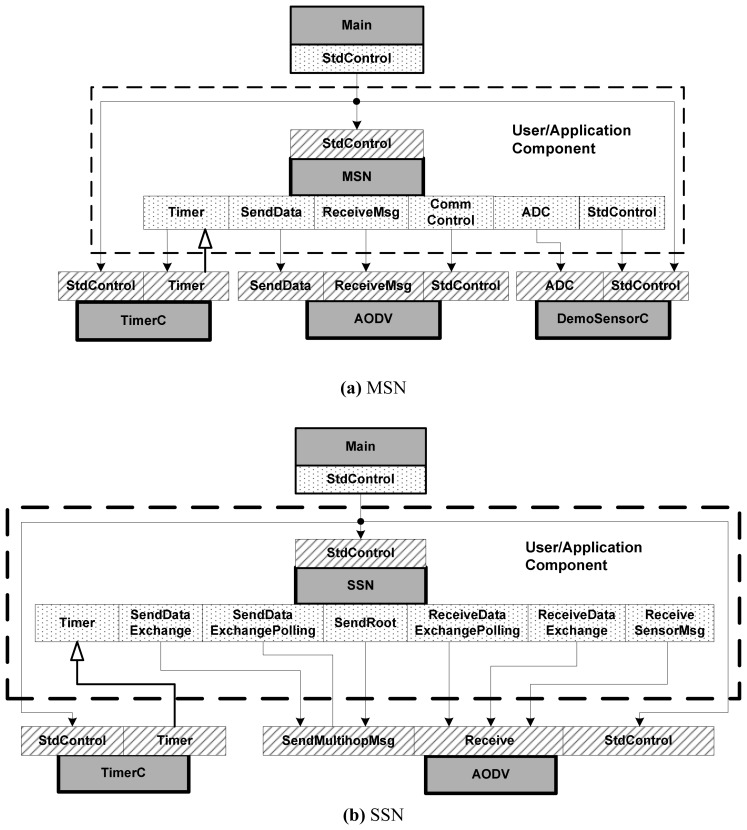
The Structure Framework of MSN and SSN.

According to the functional description, we designed MSN and SSN in TinyOS system as shown in Figure 6 (a) and (b). Here, the grey square stands for a component; the shadow square is the interface that a component provides; the dotted square is the interface that a component uses; the line with a black arrow means a command sending from the tail of the arrow to the head of the arrow; and the line with a white arrow means an event informing from the tail of the arrow to the head of the arrow.

The components that a MSN uses are Main component (which is the access point of all programs); the user/application component MSN (which executes all the functions of a MSN); the sampling timer component TimerC; the sampling and A/D conversion component DemoSensorC and the data transmission and multi-hop routing component AODV. Similarly, the components that a SSN uses are Main, SSN, TimerC (which is used for polling timing) and AODV. Here, the AODV is the acronym of Ad hoc On-Demand Distance Vector Routing [32]. This is an on-demand distance vector routing protocol which can support the multi-hop routing scheme and are widely used in the Ad hoc networks. The AODV component is provided by the TinyOS system which helps to realize the sending and receiving of the control information and data messages easily between different sensor units.

### 5.2. Visualization of System Operation

Based on the simulation platform illustrated in section 5.1, we can visualize and monitor the system operation in OMNet++ [33]. The purpose of the visualization is to investigate the initialization/ configuration of the system and the performance of the routing protocols. Besides, we need to know if the polling and data exchange messages can be transferred correctly or not, and if the distributed data mining algorithm is performed as expect.

**Figure 7.**
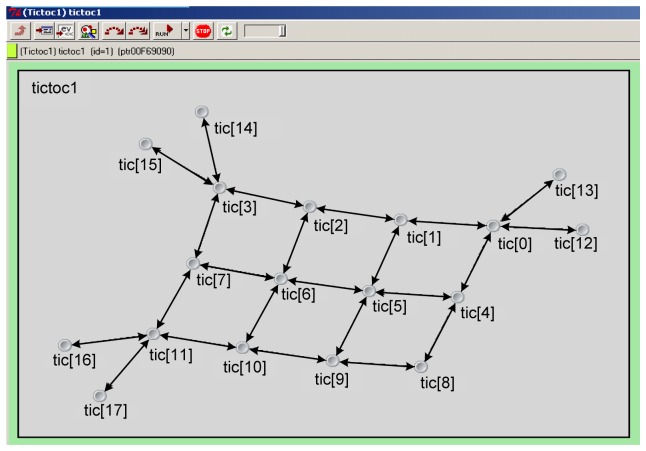
The network topology of the simulation.

The network topology of the simulation is designed as Figure 7. There are 18 sensor nodes, including 12 SSN nodes from tic[0] to tic[11] and 6 MSN nodes from tic[12] to tic[17]. Data can be sent and received in bi-directions along the edges. We use the air pollution data of four pollutants NO, NO_2_, SO_2_, and O_3_ at 1-minute intervals in urban environment from 8:00 to 17:59 within a day as the sampled data for each MSN. The total number of the dataset is 7200.

**Figure 8.**
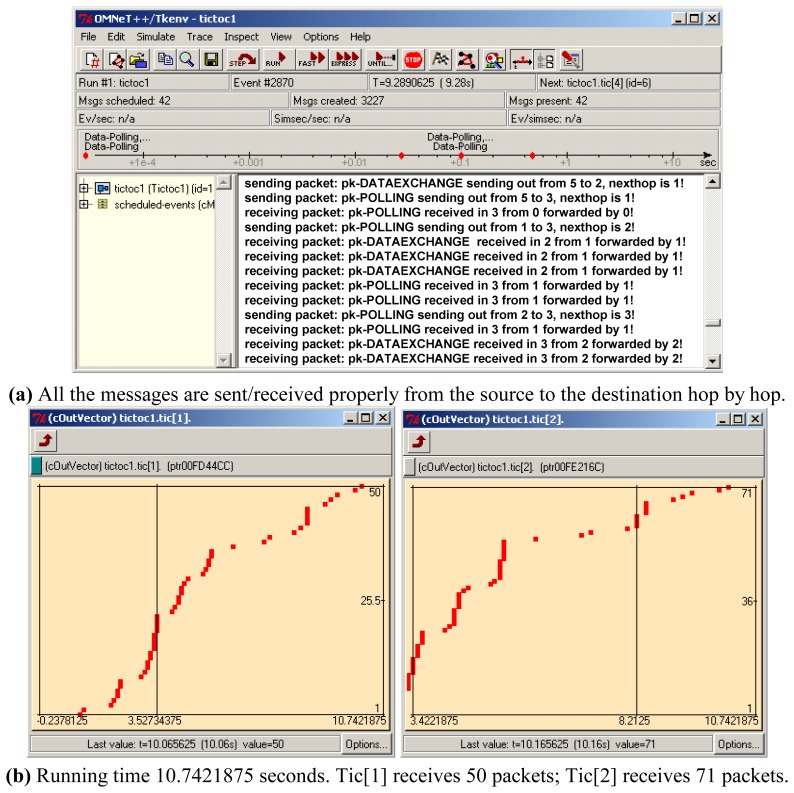
Simulation monitor results

The simulation is performed on the computer with P4 CPU, 3.2GHz main frequency. The link delay is set to be 100ms. The running details of the system can be monitored as shown in Figure 8.

After the initialization, all the nodes begin to exchange the polling and data messages. We can see from Figure 8(a) that all the messages can be sent and received between the source node and the destination node hop by hop. Figure 8(b) shows the receiving procedures of every packet in tic[1] and tic[2] within 10.7421875 seconds, while tic[1] received 50 messages and tic[2] received 71 messages. These messages include all the control messages (such as initialization, topology discovery and routing information), polling messages and data exchange messages. The receiving speeds in these nodes present an approximately linear increasing, which means the system can send and receive messages with very low collision and packet lost.

### 5.3. Operation of the Distributed Clustering Algorithm

**Figure 9.**
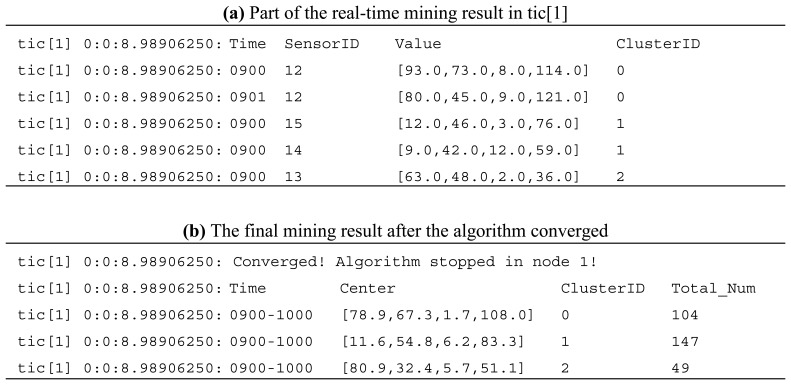
The distributed clustering algorithm test result in tic[1].

The distributed clustering algorithm test results in tic[1] are shown in Figure 9. The test dataset is the air pollution data of four pollutants NO, NO_2_, SO_2_, and O_3_. Each data record has a time stamp between 9:00 and 10:00, a sensor ID which shows the data source, and four values of the volumes of the pollutants. Here we defined the *k*-means algorithm with *k* = 3. So there will be three clusters with the cluster ID of 0, 1 and 2. Result (a) is a part of the real-time mining result in tic[1]. We can see that, in each recursive processing of the algorithm, SSN tic[1] computes the cluster centers and assigns the distributed data it gathered to a corresponding cluster. In result (b), the algorithm finishes according to the converging criteria and tic[1] gets a local clustering result. The final cluster centers and total number of data points in tic[1] are also shown in the result.

The comparison of the average clustering accuracy of the centralized and distributed clustering algorithms is shown in Table 1. For the centralized clustering algorithm, we suppose tic[8] be the sink (central point for data processing) in the topology shown in Figure 7, which means every other node sends the collected data to tic[8]. And the classic *k*-means algorithm [34] is running in tic[8] for centralized clustering. For the accuracy measurement, let *X^i^* denote the dataset at node *i*. Let $L_{km}^{i}(x)$ and *L^i^* (*x*) denote the labels (cluster membership) of sample *x* (*x* ∈ *X^i^*) at node *i* under *k*-means algorithm and our distributed clustering algorithm respectively. We define the Average Percentage Membership Match (*APMM*) as

(1) $$\text{APMM}=\frac{1}{n}\sum_{i=1}^{n}\frac{\left|\left\{x\in X^{i}:L^{i}(x)=L_{km}^{i}(x)\right\}\right|}{\left|X^{i}\right|}\times 100\%$$

Where *n* is the total number of SSNs.

For the distributed clustering algorithm, we vary the number of nodes in the Information Exchange Node Set (IENS) of each SSN from 1 to 10. Data are randomly assigned to each SSN. Table 1 shows the *APMM* results.

From Table 1 we can see that, when the number of nodes in IENS is no less than 2, in other words, when each SSN exchanges data with at least two other SSNs, the *APMM* exceeds 90%. When the number of nodes in IENS is no less than 4, the *APMM* exceeds 93%. The results are achieved under the condition of assigning the data to each SSN randomly. In reality, if the patterns of the dataset are various in different locations, the *APMM* maybe lower than the results in Table 1. In such situations, a good scheme of how to choose the nodes to construct the IENS would be very important.

## 6. Data Analysis Scenario

**Figure 10.**
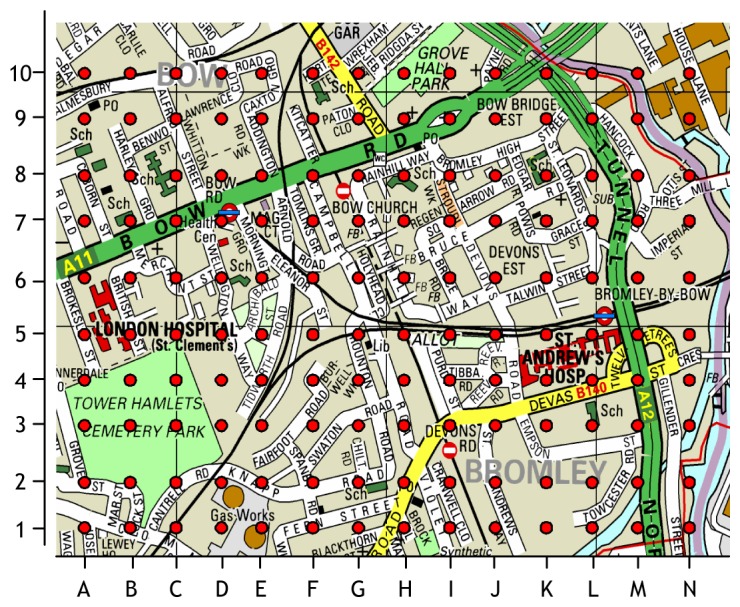
MoDisNet sensors evaluation case in an area of east London.

In this section, we present a real-time pollution data analysis scenario to evaluate the data analysis capability of MoDisNet system. This evaluation is based on the air pollution data that was generated from our former project Discovery Net which constructed a sensor grid over a typical urban area as shown in the map of Figure 10 around the Tower Hamlets and Bromley areas in east London. There are some of the typical landmarks such as the main road extending from A6 to L10, the hospitals around C5 and K4, the schools at B7, C8, D6, F10, G2, H8, K8 and L3, the train stations at D7 and L5 and Gas Works between D2 and E1. 140 sensors are distributed in this area and collect data from 8:00 to 18:00 at 1-minute intervals to monitor the pollutants of NO, NO_2_, SO_2_ and O_3_.

**Figure 11.**
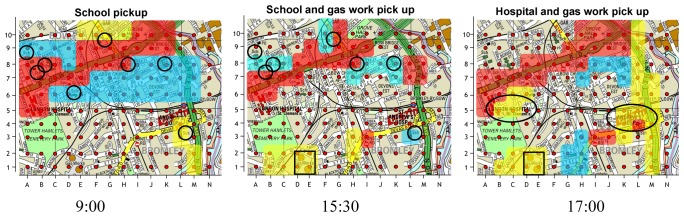
Pattern recognition for high air pollution level areas.

The air pollution analysis uses the air pollution data to give an overall understanding of the air pollution characterization within this area by running the data mining algorithm. As the Discovery Net can only classify the pollution data into several pollution levels, such as high or low, but can't tell us the distribution of different pollutants in different locations and their contributions to the pollution levels. To improve the data analysis capability, in this data analysis scenario, we use the distributed clustering algorithm to cluster the pollutants into pollution clouds which can recognize different pollution patterns. From the experimental results of Discovery Net, we pickup all the high pollution level locations at 9:00, 15:30 and 17:00 respectively to check the contribution of different pollutants (NO, NO_2_, SO_2_ and O_3_) to the pollution levels. The results are shown in Figure 11.

From the figures we can see that in the morning at 9:00, the high pollution locations are distributed around the main roads and the schools (highlighted by the circles). At 15:30, the high pollution locations are around the schools and the factory (which is a gas work and highlighted by the square). At 17:00, more pollution focuses on the main roads; the hospitals (highlighted by the ellipses) and the factory make the contribution as well. Checking the mining data set, we found the relationship between the pollution clouds, the different pollutants and pollutants' volumes, which is shown in Table 2.

This table shows that, in each time snap, there are 3 different pollution clouds: Red, Blue and Yellow. Each cloud represents a kind of combination of the 4 pollutants. For example, in the morning at 9am, the Red cloud that covers the main roads and the area around the main roads is characterized by high volumes of NO and SO_2_. Most of these pollutants are emitted by the vehicles running in the morning rush hours. According to the environmental reports, today in the UK, the road transport sector is the major source of NO_X_ emissions, especially in urban areas, contributing more than 50% to the total emission. Besides, the solid fuel and petroleum products are two main contributors of SO_2_. Herein, it is still an urgent research topic for us to reduce the transport emissions and produce clearer diesel fuel.

At 15:30 in the afternoon, the Blue clouds cover the school areas, which are in high volumes of NO_2_ and O_3_ with low volume of NO. While Yellow cloud featured by high volume of SO_2_ covers the factory area. As NO_2_ and O_3_ are all formed through a series of the photochemical reactions featuring NO, CO, hydrocarbons and PM, generating NO_2_ and O_3_ needs to take a period of time. That is the reason that the density of NO_2_ and O_3_ in the afternoon is higher than that in the morning traffic peak time.

At 17:00 in the afternoon, it seems to be the worst pollution distribution time within a day. Besides the transport emission around the roads and the factory emission, some other locations such as the hospitals contribute some kind of pollutants, including the sulphide and nitride. On the right most of Figure 11, a yellow cloud covering a main road from M1 to L10 contains high density of SO_2_, NO_2_ and O_3_. The pollution pattern is very similar to the pattern at the factory and hospital areas, but not similar to the pattern on the other main road (from A6 to K10). We investigated this area and found that, a brook flows along this area in the near east and a factory area locates on the opposite side of the brook which is beyond the scope of this map. This can explain why the pollution patterns are different on these two main roads.

## 7. Conclusions

In this paper, we have provided an overview of the urban air pollution analysis within MoDisNet project, describing the network framework, the GUSTO sensor technology, the mobile sensor grid architecture and the distributed data mining algorithm. The system can achieve a high performance based on the high quality mobile sensing capability of GUSTO sensor unit which can measure pollutants at very short intervals. Besides, with respect to the distribution of the sensors within the large area of urban environments and the data integration requirements during the transmission and analysis, the well designed e-Science Grid architecture and distributed data mining algorithm are essential for this scenario.

We are currently researching on the data fusion and aggregation technique [35, 36, 37] to improve the system performance when large amount of data are collected and transferred, especially when the third part data (including the traffic data and weather data) are imported. As discussed in section 3.3.2, the Grid architecture and multi-hop routing capability enable the MoDisNet system to implement the data fusion and aggregation. It can save the communication cost, reduce the power consumption of the sensor units, and increase the available bandwidth of the wireless channels. One attempt is to design the fusion and aggregation algorithms with the integration of novel routing protocols; another way is to analyze the pollution data collected in different locations to find the redundancy of the data, so as to decrease the number of active sensors or prolong the sample intervals. Beside, when the mobility pattern of the vehicles is taking into consideration, the data communication and analysis may face new challenges, such as noise reducing, positioning, mobile data collecting and identifying, etc.

As UK has obligations as a member of the United Nations Economic Commission for Europe (UNECE) regarding transboundary pollutants that cause harm to the environment, reducing the impact of road traffic on the environment is vital to the government and individuals. Of all emissions contributed mainly by road transport, monitoring PM_10_ and NO_X_ are currently most desired, with expectations that finer detection (e.g. PM_2.5_) will be needed in the future and also a need for more detailed monitoring of noise levels. As addressing global warming becomes more important in government policy however, local authorities are likely to be increasingly required to monitor and reduce greenhouse gas emissions in their regions. Information on greenhouse gases is therefore also needed for long term monitoring purposes with similar linkages to traffic and weather data to understand the contribution of traffic to environmental conditions.

## Figures and Tables

**Table 1.** Centralized Clustering vs. Distributed Clustering (*APMM* results).

IENS	1	2	3	4	5	6	7	8	9	10
*APMM*	84.5%	90.29%	92.48%	93.06%	93.1%	93.32%	93.43%	93.66%	94.05%	94.4%

**Table 2.** Pollution pattern analysis.

Time	Clouds	Analysis			
		NO	NO_2_	O_3_	SO_2_
9:00	Red	High	Middle	Low	High
	Blue	High	Low	Low	Low
	Yellow	Low	Middle	Low	Middle
15:30	Red	Low	Middle	Middle	Middle
	Blue	Low	High	High	Middle
	Yellow	Low	Low	Low	High
17:00	Red	Middle	High	High	Middle
	Blue	Low	Low	Middle	Middle
	Yellow	Low	Middle	Middle	High
